# Prediction of myeloid malignant cells in Fanconi anemia using machine learning

**DOI:** 10.1371/journal.pone.0340578

**Published:** 2026-01-20

**Authors:** Luis A. Flores-Mejía, Pablo Siliceo, Ulises Juárez Figueroa, Angel A. De la Cruz, Cecilia Ayala-Zambrano, Hugo Tovar, Sara Frías, Alfredo Rodríguez

**Affiliations:** 1 Departamento de Medicina Genómica y Toxicología Ambiental, Universidad Nacional Autónoma de México, México; 2 Laboratorio de Falla Medular & Carcinogénesis, Instituto Nacional de Pediatría, México; 3 Laboratorio de Citogenética, Instituto Nacional de Pediatría, México; 4 Programa de Doctorado en Ciencias Biomédicas, Universidad Nacional Autónoma de México, México; 5 Programa de Doctorado en Ciencias Biológicas, Universidad Nacional Autónoma de México, México; 6 Programa de Maestría y Doctorado en Ciencias Bioquímicas, Universidad Nacional Autónoma de México, México; 7 Computational Genomics Division, Instituto Nacional de Medicina Genómica (INMEGEN), Mexico City, Mexico; Faculty of Medicine of Tunis, TUNISIA

## Abstract

Fanconi anemia (FA) is an inherited bone marrow failure syndrome with cancer predisposition. Most FA patients develop aplastic anemia during childhood and have an extremely high cumulative risk to develop cancer during their lifespan. Myeloid malignancy is one of the main neoplastic risks for patients with FA, including high-risk myelodysplastic syndrome (MDS), recently renamed as myelodysplastic neoplasm, and acute myeloid leukemia (AML). Although bone marrow transplantation is the treatment of choice for FA patients that develop aplastic anemia, patients with a more stable bone marrow remain not transplanted and at a high risk of presenting MDS/AML, these patients therefore should be monitored for appearance of myeloid malignant clones. Markers for an as-early-as-possible identification of emerging myeloid malignant cells are needed for the monitoring of patients with FA, since quick medical action after detection of neoplastic transformation is needed. In this work we have developed a deep neural network (DNN) model that was trained with publicly available single cell RNA-seq (scRNA-seq) datasets of patients with AML and used to predict the presence of AML-like cells in scRNA-seq datasets obtained from bone marrow samples of patients with FA. The predictor displayed high sensitivity, specificity, and accuracy for the detection of single-cell resolution myeloid malignant transcriptional profiles. Functional analyses of the predicted-AML cells from FA patients showed enrichment of lympho-myeloid-primed progenitor (LMPP) and granulocyte-monocyte progenitor (GMP) populations, as well as transcriptional profiles associated with malignant transformation. Cues of immune evasion were also detected using single cell pathway analysis (SCPA) and cell-cell communication profiles.

## Background

Inherited bone marrow failure syndromes (IBMFS) are rare diseases characterized by physical abnormalities, and an exacerbated risk to develop bone marrow failure (BMF) [1,2]. Among them, Fanconi anemia (FA) is the most frequent, with a global occurrence estimated to be 1 in every 160,000–360,000 individuals, with a carrier frequency of 0.3% and a higher prevalence in populations with high consanguinity rates [3,4]. Patients with FA have inherited defects in the FA/BRCA pathway, responsible for the repair of DNA interstrand crosslinks (ICLs) [5–7].

Individuals with FA exhibit an extremely high predisposition, 500–1000 times higher than that in age-matched peers to develop squamous cell carcinomas in the oral cavity and anogenital region [7,8], and a significantly increased lifetime risk to develop myeloid malignancies, such as myelodysplastic neoplasm (MDS, up to 6000-fold increased risk) and acute myeloid leukemia (AML, approximately 700 times higher than that in the general population) [7,9,10].

Given the complex aetiology of FA, multiple therapeutic approaches are implemented for treating BMF in these patients, including administration of androgens and hematopoietic growth factors, as well as hematopoietic stem cell transplantation (HSCT) [4,11]; the last one remains the only curative treatment for BMF and MDS/AML in FA, but it is not exempt of serious complications, including graft-versus-host disease and secondary malignancies [12]. Moreover, early clonal evolution toward MDS or AML often precedes clinical symptoms, making early detection critical but challenging [13].

MDS is a clonal hematopoietic neoplasm characterized by bone marrow (BM) dysplasia, compromised hematopoiesis and variable risk of progression to AML. One out of three patients diagnosed with MDS will progress to AML, characterized by an increased percentage (>20%) of myeloid blasts in the BM. Despite the high risk of leukemic transformation in FA patients, current monitoring protocols vary between institutions, and the optimal timing or tools for early detection remain under discussion [6,9,14,15].

Common BM surveillance techniques include GTG karyotype and fluorescent *in situ* hybridization (FISH). Among cytogenetic abnormalities, duplication of chromosome 3q (3q+), deletion of 7q (7q−), and monosomy 7 (−7) are considered high-risk markers for progression to MDS and AML in FA patients [9]. These aberrations not only mark the emergence of MDS but also help identify cases at highest risk for rapid leukemic evolution. Hence, high-risk MDS and AML with MDS-related changes are increasingly recognized as a continuum of disease progression rather than discrete clinical entities [15].

Presence of MDS and AML clones is an indicator for BM transplantation (BMT) in patients with FA, making timely detection of malignant clones a critical step to expedite the BMT preparatory regime [5,16,17]. Although BM karyotype and FISH are highly reliable techniques for the detection of malignant clones, they are time-consuming. In patients with the highest risk of neoplastic transformation, such as patients with FA, the earliest possible detection of abnormal clones is of the utmost importance since rapid evolution of malignant clones in these patients is commonly observed [5,18].

Recent single-cell RNA sequencing (scRNA-seq) technologies have increased our understanding of the transcriptional programs of multiple cancer types at unicellular resolution [19–21]. The advent of single-cell profiling and publicly available AML datasets [22,23] can be exploited to understand the spectrum of the MDS-AML myeloid malignancies.

Artificial intelligence (AI) has gained relevance for analyzing large and complex multidimensional datasets. In the field of AI, machine learning encompasses multiple pattern recognition algorithms used for fitting predictive models to data and/or identifying informative groupings within data [24]. Artificial neural networks are models that consist of multi-layered interconnected nodes that, by mimicking the neuronal connectivity of biological brains, learn hierarchical features of data. Each node, situated within a layer, symbolizes a weighted computation of a vector of variables, learning key aspects of the data and transmitting “learned” information between nodes from the network’s input, hidden, and output layers [24].

This deep learning approach allows the utilization of the above mentioned large and complex cancer scRNA-seq datasets to train machine learning algorithms with the capacity to predict malignant cells from complex cell populations, such as the BM of cancer predisposition syndromes, including FA.

In this work we developed and trained a multi-layer deep neural network (DNN) model for predicting and identifying cells with AML-related transcriptional profiles in scRNA-seq datasets from the BM of patients with FA. In this model, the architecture of the DNN consisted of an input layer corresponding to the normalized gene expression matrix, followed by multiple hidden layers with nonlinear activation functions (ReLU), and a final output layer producing binary classification scores [25]. The model was trained using supervised learning with labelled data derived from known AML and healthy scRNAseq profiles, and optimized using a cross-entropy loss function via backpropagation and stochastic gradient descent. Regularization techniques such as dropout and early stopping were employed to prevent overfitting and improve generalization. The predicted-AML cells were found enriched in the lympho-myeloid-primed progenitor (LMPP) and the granulocyte-monocyte progenitor (GMP) compartments, displaying gene expression profiles compatible with malignancy. We further analyzed the gene expression profile of these predicted AML cells and propose some markers for its identification.

## Methods

### Classifier

#### Data summary.

To train the model with a solid compendium of single-cell RNAseq data comprising transcriptomic profiles of AML cells, we downloaded count matrices from 16 patients with a confirmed diagnosis of AML, as well as 2 healthy donors with a normal bone marrow, according to van Galen *et al* (2019, GEO access number GSE116256) [23]. For our query dataset, on the other hand, we used publicly available raw sequencing reads from 6 patients with a confirmed diagnosis of FA and 4 healthy donors with a normal bone marrow, according to Rodríguez *et al* (2021, GEO access number GSE157591) [26].

### Processing of single-cell expression matrices

Raw sequencing data from Rodríguez *et al* (2021, GEO access number GSE157591) [26] was processed with the Cell Ranger pipeline (v9.0.1) for alignment with the GRCh38 reference genome, barcode demultiplexing, and UMI counting. The resulting count matrices, along with the ones downloaded from van Galen *et al* (2019, GEO access number GSE116256) [23], underwent standard quality control filters using Seurat (v5.2.1) (min.cells = 3, min.features = 200, max.features = 5000, mt.percent <10%).

### Model input formatting

To generate a single dataset to train and validate the model, datasets corresponding to healthy donors and AML patients from van Galen *et al* (2019, GEO access number GSE116256) along with healthy donors from Rodríguez *et al* (2021, GEO access number GSE157591) were first merged into a single Python (v3.13.3) object composed of a matrix with cells as rows and genes as columns. Only intersecting genes between the two datasets where kept, these shared genes were taken as the input variables for the model.

The combined dataset was transformed into an annotated object using the *AnnData* function from Scanpy (v1.11.1), with observations as cells, shared genes as variables and annotations as metadata. Annotations from the van Galen *et al* (2019) dataset (malignant and healthy) were already available from [23], meanwhile all cells from healthy donors from the Rodríguez *et al* [26] dataset were annotated as “healthy”.

**Fig 1:**
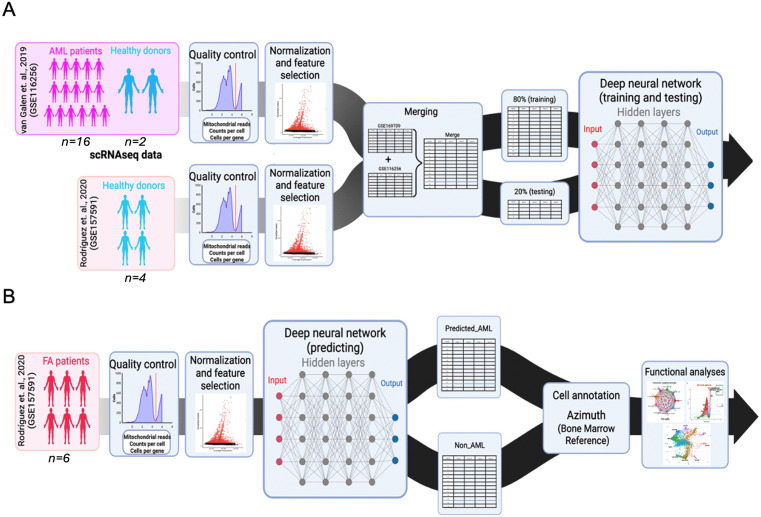
Development of a single-cell resolution deep neural network (DNN) predictor for AML cells in patients with FA. **(A)** Workflow illustrating the preprocessing of publicly available single-cell RNA sequencing (scRNA-seq) datasets from AML patients and healthy donors. A deep neural network (DNN) model was trained to identify AML cells (predicted_AML, and non_AML). The training set was composed of 16 patients with AML and 2 healthy donors from the van-Galen et al. (2019) dataset (GSE116256), as well as 4 healthy donors from the Rodríguez et al. (2021) dataset (GSE157591). The combined datasets were split into two sets, 80% of cells were used for training and 20% were used for validation. **(B)** The trained model was used to predict AML-like cells, i.e., those with gene expression profiles similar to AML cells, in a dataset composed of 6 patients with FA from the Rodríguez et al. (2021) dataset (GSE157591). Cell annotation and functional analyses were performed in the classified cells.

RNA counts were normalized using the Scanpy (v1.11.1) function *normalize_total* (target_sum = 1e6) and later log-transformed using the *log1p* function (default parameters were used). To format the data into the training and testing sets for the model, annotations were categorically encoded into binary values (healthy = 0, malignant = 1) and an array with those values was created using the *array* function from Numpy (v2.2.5). Both binarized annotations and their corresponding RNA count values were randomly divided into the training sets, accounting for 80% of the data, and the testing sets, with the remaining 20%, using the scikit-learn (v1.10) function *train_test_split* (shuffle = T) (**Fig 1A and 1B**).

### Building and testing the model

A deep neural network (DNN) model was built using the deep learning API from Keras (v3.9.0). The model consisted in a sequential layer-based model with 3 hidden layers (Layer 1 = 1000 nodes, Layer 2 = 800 nodes, Layer 3 = 50 nodes) with a sigmoidal type of activation, while the output layer used a softmax activation function. The model was configured using the *model.compile* function (loss = ‘sparse_categorical_crossentropy’, optimizer = Adam(), metrics=[“accuracy”]). Finally, the model was trained with the 20% of the combined datasets with the *model.fit* function (batch_size = 120, epochs = 8).

### Predicting malignant cells

The query dataset, consisting of the FA patients [26], was formatted as described earlier and their cell types were predicted with the DNN model using the *model.predict* function, taking the normalized RNA counts as input. Output predictions were renamed from healthy and malignant to non_AML and predicted_AML, respectively.

#### Cell Annotation, StemNet visualization and differentially expressed genes (DEG) evaluation.

After obtaining predictions, the dataset was transferred to an R environment, where a Seurat object was created using the *CreateSeuratObject* function. The object consisted of previously normalized RNA counts across all cells and predictions as meta.data.

Following this, the Azimuth (v0.5.0) algorithm, an automated reference-based approach for single-cell annotation, was applied to the Seurat object using the Human – Bone Marrow reference. This reference includes 297,627 bone marrow cells from 39 donors and three different studies [27–29], as well as the Human Cell Atlas Immune Cell Census.

STEMNET (Velten et al., 2017) was employed to reconstruct the differentiation trajectory, specifically a gradient commitment toward the lymphoid-myeloid and megakaryocyte-erythroid lineages.

Differential expression analysis between groups was conducted using the DESeq2 R package (version 1.42.0). Raw count data were normalized, and dispersion estimates were calculated by applying the DESeq function. Differentially expressed genes (DEGs) were identified based on an adjusted p-value (Benjamini-Hochberg corrected) threshold of 0.05. To focus on genes with the highest variability, the top 70 genes ranked by variance across samples were selected for heatmap visualization using the pheatmap package (version 1.0.12). Volcano plots were generated utilizing the EnhancedVolcano package (version 1.15.4), plotting log2 fold change (log2FC) against the adjusted p-value to visually represent significantly up- and down-regulated genes. Stringent cutoffs were applied for volcano plot annotation, setting adjusted p-value < 1e-5 and |log2FC| > 1. Key genes of biological relevance, including *TP53* and *MYC*, were highlighted. Custom color schemes were employed to distinguish expression levels, and connectors were drawn to enhance label clarity.

#### Single cell pathway analysis (SCPA).

SCPA was conducted in RStudio (v2024.12.1 + 563) using the SCPA package (v 1.6.2), which involved extracting log1p normalized data from each relevant population. The pathways used in the analysis were generated from the publicly available molecular signatures database using the msigdbr package (v10.0.2) within R. Comparisons were performed using the *compare_pathways* function within SCPA, with the only inclusion criteria being gene sets with 15–200 genes [30]. Data processing and visualization was carried out using the Seurat (v5.2.1), ggplot2 (v3.5.2), and ComplexHeatmap(v2.18.0) R packages.

Subsequently, expression levels of genes codifying for cell surface proteins and soluble factors, including immunomodulatory proteins and growth factors, were evaluated across cell types. The SCpubr package (v1.2.0) was used to generate customized boxplots from the normalized expression data of each population. Pairwise comparisons per marker were conducted among healthy cells, FA Non-AML, and FA Predicted-AML cells using the Wilcoxon rank-sum test as implemented in SCpubr. Markers evaluated include LGALS9, CD200, CD74, IL-16 among others. The boxplots were generated without silhouette plots and significance annotations were displayed to prioritize clarity in the visualization of expression distributions. These analyses enabled the assessment of differential expression of specific surface proteins and soluble factors relevant to immune regulation and disease progression.

### Code availability

The relevant code supporting the findings of this article is available in the following github repository https://github.com/BMF-CP-Lab/DNN-AML-MDS-classifier

### Ethics statement

Publicly available pre-processed scRNAseq datasets were retrieved from public repositories, including van Galen et al (2019, GEO access number GSE116256) [23] and Rodríguez et al (2021, GEO access number GSE157591) [26]. All data were fully anonymized, and the authors did not have access to patient’s identity. This project was approved by the Institutional Review Board (IRB) from the National Institute of Pediatrics in Mexico, under approval number: 2023/003. This IRB is registered by the U.S. Department of Health and Human Services (HHS) under IRB number: IRB00013674.

## Results

**Fig 2:**
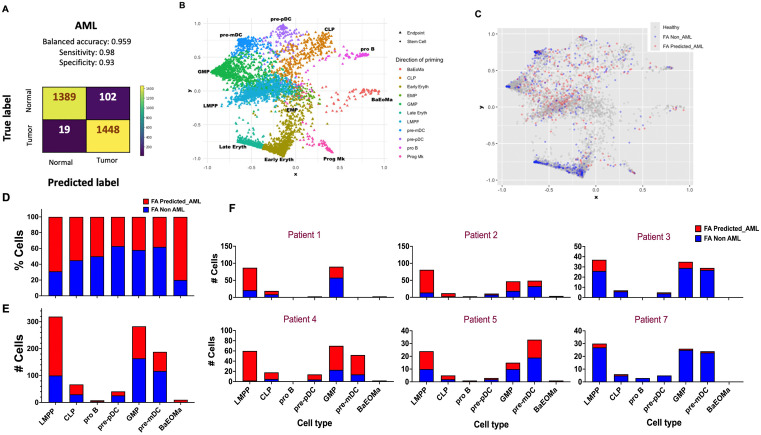
Predicted AML cells are more abundant in the lymphomyeloid lineage of patients with FA. **(A)** Confusion matrix of the AML DNN predictor model. **(B)** StemNet plot showing the trajectory of differentiation of HSPCs from FA patients. The GMP, CLP and LMPP progenitors are the more undifferentiated cell types (as shown by the diamonds in the centre of the plot). **(C)** StemNet plot projecting the predicted-AML cells in FA patients (red dots). The FA predicted-AML cells are mainly found in the undifferentiated lymphomyeloid compartments (LMPP, GMP and CLP), as indicated by the centrally located red dots in the plot. **(D)** Bar plot showing the percentage of predicted-AML cells (red stacked bar) per cell type in samples from FA patients. **(E)** Bar plot showing the number of predicted-AML cells (red stacked bar) per cell type compartment in samples from FA patients. **(F)** Bar plots showing the number of predicted-AML cells (red stacked bar) per cell type compartment per FA patient.

Using bone marrow derived scRNAseq datasets from healthy donors and patients with AML, we developed and trained a DNN model that predicts, at the single cell level, cells with AML transcriptional profiles (**Fig 1A**). This model was used to predict the presence of cells with transcriptional profiles resembling AML cells in the BM of patients with FA (**Fig 1B**) [23]. Our DNN AML classifier displays 96% accuracy, 98% sensitivity and 93% specificity in the prediction of cells with AML-like transcriptional profiles (Fig 2A).

Using Azimuth [27–29] as reference, we annotated the scRNA-seq dataset from the BM of patients with FA, and obtained different cell types, including Hematopoietic Stem Cells (HSC), Erythroid Megakaryocyte Progenitor (EMP), Lymphoid Primed Multipotent Progenitor (LMPP), Common Lymphoid Progenitor (CLP), Granulocyte Monocyte Progenitor (GMP,) Early Erythroid, Late Erythroid, Progenitor B (pro B), Precursor Plasmacytoid Dendritic Cell (pre-pDC), Precursor Myeloid Dendritic Cell (pre-mDC) and Basophil-Eosinophil-Mast Progenitor (BaEoMa). Then we performed a StemNet representation analysis to display the different progenitors and their maturation state (Fig 2B). We visualized the distribution of the predicted_AML, non_AML, and healthy cells from Rodríguez *et al* [26] and observed that most of the FA predicted_AML cells appear in the LMPP compartment and were less differentiated (at the center of the StemNet plot) (Fig 2C). We then calculated the percentage of predicted_AML cells per cell type (Fig 2D), the number of predicted_AML cells per cell type (Fig 2E) and the number of cells per cell type and per patient (Fig 2F), in every case comparing with respect to the FA Non-AML cells.

After identification of the FA predicted_AML cells we used pseudo-bulk RNA seq analysis to compare the gene expression profile of the FA predicted_AML cells against healthy cells, the remaining FA non-AML cells, and against AML cells. Interestingly, differentially expressed genes (DEG) obtained through this analysis resulted in the distinction of three gene modules corresponding to the different cell identities. *Module A* is composed of genes expressed only in healthy cells and downregulated in all FA cells as well as in all AML cells. *Module B* is a set of genes expressed only in the FA non_AML cells and whose expression is lost in the FA predicted-AML cells. Finally, *Module C* is composed by genes that are down-regulated in healthy and FA non-AML cells, that start to gain expression in the FA predicted-AML cells and are full blown activated in the AML cells, suggesting an activation gradient as the cells progress from non-malignant towards AML (**S1A Fig**). Module score analysis with single cell resolution confirmed the downregulation of module A genes in AML cells and in the FA predicted-AML cells (**S1B Fig**). Module A is composed of potentially tumour suppressor genes like *PCDH9* [31,32], aging-related genes like *ATP6V1G3* [33,34], genes involved in cell cycle regulation and cancer invasiveness like *PKP2* [35], long noncoding RNAs like *HCN3* [36], *HIST2H2AA4* [37], *Six3os* [38], and *LINC01173* related with homeostasis maintenance [39] and cancer inhibition [34,40,41], pseudogenes in this module with an undefined functions include *RN7SL668P*, *RNA5SP68*, *RNU6ATAC27P, RP11-78H24.1* and *RP11-696F10.1*.

**Fig 3:**
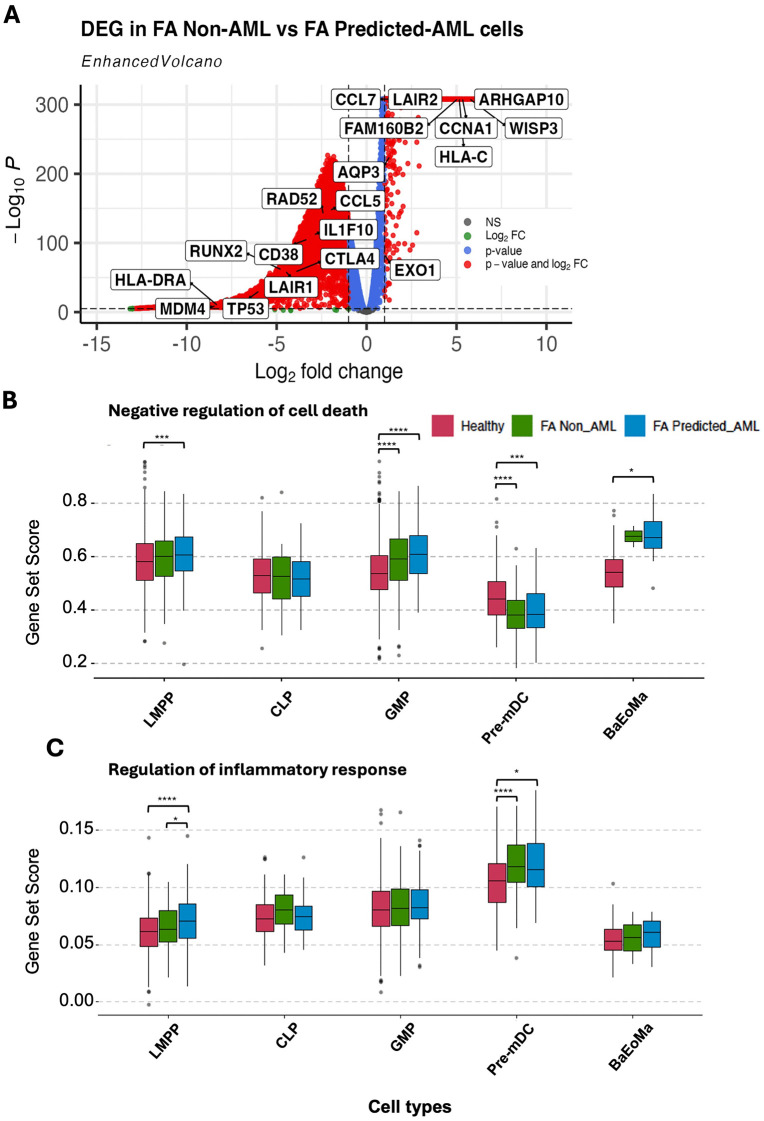
Gene expression profile of the predicted AML cells from FA *patients.* **(A)** Volcano plot showing DEG between the FA predicted_AML cells and the FA non-AML cells; pCutoff = 1e-5, FCcutoff = 1. **(B)** Single-cell pathway analysis (SCPA) showing enrichment of negative regulators of cell death pathways, specifically in the FA predicted_AML cells. **(C)** SCPA showing enrichment of inflammatory response pathways in sub-compartments of the FA predicted_AML cells.

We also assessed DEG between the FA predicted_AML cells in comparison to the FA FA non_AML cells. Of note, we obtained a distinct gene expression profile, including increased expression of *WISP3,* a gene that has been previously associated with aggressive inflammatory breast cancer and breast cancer metastasis [42–44], and *CCNA1* (Cyclin A1) a canonical cyclin that promotes S and G2 phase progression, previously reported to be overexpressed in up to 82% of AML cells [45–48]. We noted also downregulation of immune inhibitory molecules, specifically *CTLA4* and *LAIR1* (Fig 3A).

Later, using Single Cell Pathway Analysis (SCPA) and GO (Gene ontology) terms (Biological Processes), we aimed to identify coordinated transcriptional changes in biological pathways of interest. The pathway “*Negative regulation of cell death*” (gene list shown in the **S1 Table**) was found to be more active in the predicted_AML cells, and more prominently in the LMPP and GMP sub-compartments (Fig 3B). The “*Regulation of the inflammatory response”* was another pathway (gene list shown in the **S2 Table**) active in the FA predicted_AML cells, specifically in the LMPP, pre-mDC and BaEoMa subcompartments (Fig 3C). Importantly, promotion of an inflammatory environment is among the most reported mechanisms driven by malignant cells to promote their development and proliferation.

Another mechanism relevant to FA, particularly described in the Japanese population, is the aldehyde degradation deficiency syndrome, in which *ALDH2* provides a critical compensatory role in detoxifying formaldehyde when *ADH5* is deficient [49]. Interestingly, our expression analysis revealed upregulation of both *ALDH2* and *ADH5* in FA_predicted_AML cells compared with FA_non-AML and healthy cells (S3 Fig). These transcriptional patterns suggest that the predicted AML-like cells may gain a survival advantage under aldehyde-induced stress in the bone marrow microenvironment.

**Fig 4:**
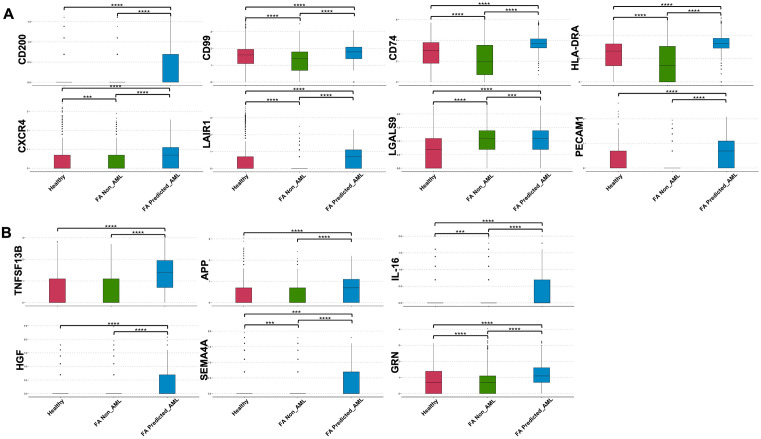
Overexpression of potential cell surface markers and soluble factors in the FA-predicted AML *cells.* **(A)** Boxplots showing overexpression of genes codifying for potential cell surface markers in the FA predicted_AML cells in comparison to healthy cells and other FA cells. **(B)** Boxplots showing overexpression of genes codifying for soluble factors in the FA predicted_AML cells in comparison to healthy cells and other FA cells. Wilcoxon rank-sum test was performed for comparisons.

We next explored the expression of potential surface markers and soluble factors differentially expressed by the FA predicted_AML cells in comparison to the FA non_AML and Healthy cells. We obtained 11 potential surface markers significantly upregulated in the FA predicted_AML cells including CD200, CD99, CD74, HLA-DR/DP/DQ, CXCR4, LAIR1, L-Selectin, P-Selectin, Galectin-9 and PECAM-1 (Fig 4A), previously reported by others authors as surface markers in different cell types [50–60] or even to identify leukemic cells [50,52,55,61–64]. We obtained also 6 potential soluble factors overexpressed by the FA predicted_AML cells, including TNFSF13B, APP, IL-16, HGF, Pro-granulin and Semaphorin-4 (**Fig 4B).** TNFSF13B (BAFF), IL-16, HGF and progranulin are known to modulate the immune microenvironment, cell survival, and inflammatory signalling [65–70]. During the progression to acute myeloid leukemia (AML), dysregulated expression or secretion of these soluble factors can contribute to immune evasion, support leukemic cell proliferation, and remodelling of the bone marrow niche favouring malignant hematopoiesis [70–73]. The soluble nature of these molecules allows them to have long-range effects, amplifying systemic effects that may further disrupt hematopoietic homeostasis and promote different disease manifestations or symptoms [74].

**Fig 5:**
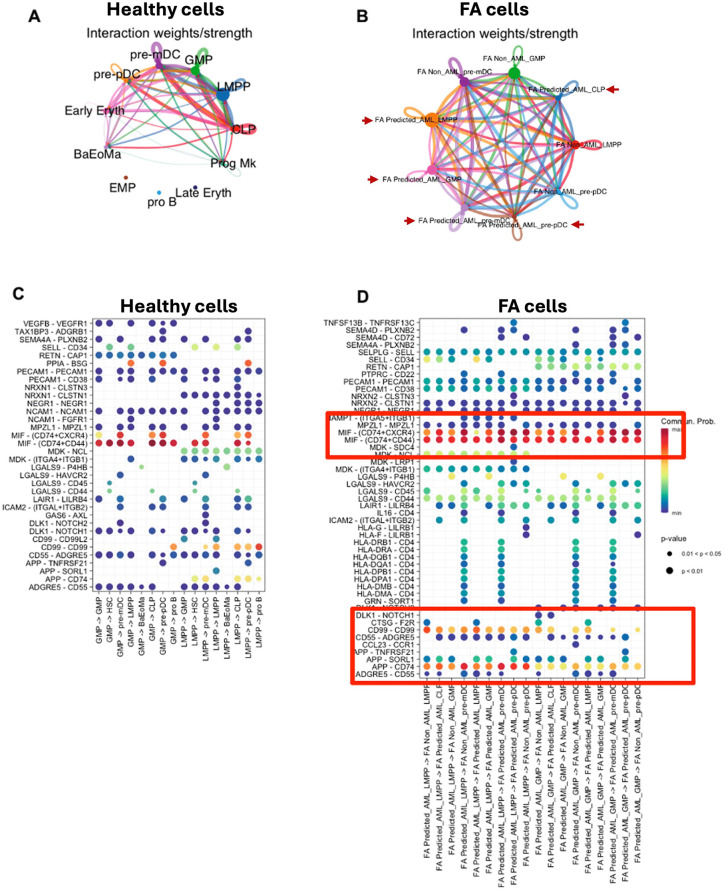
Analysis of cellular interactions between predicted_AML cells and non-malignant cell types in patients with FA. **(A)** Net Visual circle showing the interactions among healthy progenitors (LMPP and GMP) and other cell types. **(B)** NetVisual circle showing interactions among the FA predicted_AML cells (LMPP and GMP) and other cell types. The predicted malignant cells are indicated with a red arrowhead **(C)** NetVisual Bubble plot showing the signaling pathways involved in cell interaction among healthy progenitors (LMPP and GMP) and the other cell types. **(D)** NetVisual Bubble plot showing the signaling pathways involved in cell interaction among the predicted AML cells (LMPP and GMP) and the other cell types.

After identifying differential activation of signalling pathways in the FA predicted_AML cells, we evaluated whether these cells could be communicating or interacting with other cell types in the scRNA-seq dataset of FA patients. Using CellChat [75] we inferred interactions occurring among cell types and observed that the predicted_AML LMPP and GMP cell types, were the main interactors with other cell types classified as FA non_AML (Fig 5A **and 5B**).

One of the most enriched pathways of intercellular communication is the MIF- (CD74 + CXCR4) pathway (Fig 5C and 5D). MIF-CD74 interaction triggers the activation of pro-survival and proliferative Akt and ERK pathways, both important in tissue repair [76]. Also, this specific interaction has been shown to regulate tumour progression and determines patient’s outcomes in advanced melanoma and tumorigenesis [77,78]. Another enriched pathway was the CD99-CD99 pathway. CD99 is a molecule involved in crucial biological processes, including cell adhesion, migration, death, differentiation and diapedesis [79]. CD99 influences processes associated with inflammation, immune responses and cancer, including lymphoma/leukemia [80] and myeloid malignancies [52]. Finally, the APP-CD74 pathway appears increased in the FA predicted-AML cells, this pathway has been implicated in the production of beta amyloid proteins, but recent studies have reported this interaction to be associated with malignancy, including melanoma and adenoid cystic carcinoma [81,82]. Altogether, these results suggest that the predicted_AML cells, mainly LMPP and GMP sub-compartments, are activating potential early mechanisms associated to malignancy (Fig 5C **and 5D).**

Although our DNN model was trained on scRNA-seq datasets and we explored its potential to flag cells exhibiting transcriptional features associated with AML, the underlying technology imposes important limitations. The 10x Genomics 3′-capture scRNA-seq platform is optimized for transcriptomic profiling and provides restricted, non-uniform coverage of transcripts, preventing reliable detection of pathogenic variants or somatic mutations—particularly in genes with low or variable expression. [83]. Consequently, mutation-level resolution is beyond the capability of our current model. Accurate identification of genomic alterations, including those relevant to leukemic progression, would require complementary DNA-based approaches such as whole-genome or whole-exome sequencing.

## Discussion

FA is a chromosome instability and cancer predisposition syndrome, with an exacerbated risk to develop MDS and AML. We therefore rationalized that cells with gene expression profiles similar to AML could be found in the BM of FA patients even at pre-clinical stages, and that such cells could be detected using AI tools applied to scRNAseq datasets. In this work, using a DNN model we first aimed to predict and identify cells with gene expression profiles similar to *bona-fide* AML cells, and subsequently we analysed their gene expression profile to identify potential cell surface markers and infer how these cells interact with other cells in the BM microenvironment.

Very importantly, the AML-like cells predicted by our DNN model in FA patients were enriched in the LMPP and GMP compartments, suggesting a very primitive identity and a transcriptional profile that resembles physiological primitive progenitor cells. Of note, others have proposed that these are important compartments for the origin of myeloid malignancy [84]. Our model did not predict AML cells in the HSC compartment, which is probably due to the fact that FA patients have very few of these primitive cells, and therefore their capture with microfluidic single cell technologies was scarce.

Most of the predicted-AML cells were detected in four out of six FA patients (Fig 2F). In patient no. 4, BM cytogenetics at the time of scRNAseq detected a clone with chromosome 7q deletion. This chromosome abnormality is well-known to have a high negative predictive score [16], and this sole abnormality places the patients in the high-risk AML group with worst prognosis [85]. Recent work has found that 7q loss is a common event during the carcinogenesis of FA patients towards AML [17]. In patient no. 1, mildly dysplastic megakaryocytes were detected during routine BM examination, but BM karyotype was reported as normal. Patients no. 2 and no. 5 were also patients with predicted AML cells; however, no cytogenetic clones nor morphological changes were detected in their clinical routine at the moment of scRNAseq. The prediction of AML-like cells in these three patients highlights the relevance of searching novel ways, beyond conventional karyotype and FISH, to identify malignant progression. In patients no. 3 and no. 7 a negligible number of malignant cells was predicted, interestingly however, patient no. 3 was previously found to have a clone with chromosome X trisomy, an abnormality that has not been linked to MDS nor AML in FA [16,17], and might therefore not be of relevance for malignant transformation.

Gene expression analysis gave us a broad idea on the potential cellular mechanisms setting the predicted malignant cells apart from healthy cells. Interestingly, the expression profile of the predicted_AML cells suggests transformation towards malignancy, including changes in immune modulation (downregulation of the immune inhibitory molecules *CTLA4* and *LAIR1*), and changes in molecules associated to tumour progression (increased expression of *CCNA1*, *HLA-C* and *WISP3)* (**S1 Fig** and Fig 3A). Interestingly, others have reported these molecules as onco-therapeutic targets [45,86–89].

Our gene expression analysis concurs with previous reports, where overexpression of *CD74, CTLA-4, HLA-C, CD79A, IRF5* and *LAG3* has been associated with AML and other types of cancer, indicating their potential participation in tumour development, either as tumour initiators or as immunological checkpoint modulators that allow malignant progression [76,77,90–95]. Interestingly, upregulation of CD200, CD99 and PECAM1 in the FA predicted AML cells, in comparison to Healthy or FA non_AML cells (Fig 4A), opens the possibility to discover novel AML-associated markers, especially in the FA context.

SCPA analysis showed increased activation of anti-cell death mechanisms and differential activation of several inflammatory pathways in the FA Predicted_AML cells in comparison to the Healthy or FA Non_AML cells (Fig 3B**-3C**); highlighting the relevance that inflammation has in these patients, as the absence of *FANC* proteins leads to increased ROS levels, inflammasome activation and production of inflammatory cytokines [96,97].

Inferring how the predicted-AML cells interact with the rest of the BM cell populations is now possible with tools such as CellChat [75], which allows to explore the interactions among the FA predicted malignant LMPP and GMP cells and the rest of the cells [75]. In this analysis, the main pathway predicted to mediate communication between the FA predicted_AML and the remaining FA non_AML cells is the MIF-CD74 pathway. This pathway is important in the protection against injury and promoting healing in different parts of the body, but also has been reported in some types of cancer such as adenoid cystic carcinoma and melanoma [76,77,81,82]. Communication through the CD99-CD99 pathway was also detected. CD99 has been found to be relevant in lymphoma, leukaemia and myeloid malignancies [98,99]. This pathway is particularly interesting since the expression of CD99 in T cells is sought to detect minimal residual disease in acute lymphoblastic leukemia [80]; and some clinical trials propose CD99 as a therapeutic target in AML [52,100].

To the best of our knowledge, this is the first effort in which publicly available scRNAseq datasets are leveraged for training a machine learning predictor aiming to identify malignant cells in cancer prone bone marrow failure syndromes. This analysis provides insights into the identification of markers for early detection of myeloid malignant cells in the BM of patients with FA. Based on our results we aim to further characterize these AML_predicted cells and propose potential therapeutic strategies that target these malignant cells before full-blown AML occurs.

Our study has limitations. We rely on publicly available scRNA-seq data and their associated metadata, therefore we could not directly correlate our findings with the longitudinal clinical follow-up of the FA patients. The current lack of access to updated or extended clinical outcomes limits the ability to assess the predictive value of the identified AML-like cell populations in disease progression or relapse for these specific patients; however prospective search of AML-cells with markers derived from our predictions are warranted.

Our analyses are also constrained by the technical properties of the 10x Genomics Chromium 3′-end scRNA-seq platform, which captures only the terminal portion of transcripts and provides limited sequencing depth. As a result, full-length coverage of FA genes and cancer-associated genes is not achievable, precluding reliable detection of pathogenic germline variants, secondary somatic mutations (e.g., in *TP53*), or complex cytogenetic abnormalities. Similarly, the sparsity and dropout inherent to this technology limit the sensitivity of CNV-inference tools and restrict the model’s ability to resolve genotype- or population-specific effects. These constraints underscore that our DNN predictions reflect transcriptional consequences rather than direct genomic alterations and highlight the need for future integration of complementary single-cell DNA or full-length RNA sequencing modalities.

## Conclusion

In this work we implemented a DNN machine learning algorithm that was trained using publicly available scRNA-seq datasets for the detection of AML cells. Using this algorithm, we predicted the presence of AML cells in scRNA-seq datasets from the BM of patients with FA. The predicted_AML cells were found enriched in the LMPP and GMP hematopoietic compartments and have gene expression profiles compatible with malignancy. Further experimental approaches that confirm the identity of these predicted malignant cells are warranted.

## Supporting information

S1 Fig Gene modules provide identity to healthy HSPCs, AML cells and FA cells. **(A)** Heatmap of differentially expressed genes, identified through pseudo-bulk analysis of the scRNAseq datasets, among healthy cells, FA non-AML cells, FA predicted-AML cells and AML cells. Genes that allow identification of cell types are classified in modules. **(B)** Module score analysis using scRNAseq data showing average expression of gene modules per cell type.
(TIFF)

S2 Fig Expression of FA genes with respect to the mutated FANC gene in FA patients. **(A)** Bubble plot showing the average expression of the FA pathway genes per cell type, dividing FA patients according to their germinal inactive gene.
(TIFF)

S3 Fig Increased expression of ALDH2 and ADH5 in the FA predicted-AML cells. **(A)** Boxplots showing increased expression of ALDH1 in the FA-predicted AML cells in comparison to healthy and FA non AML cells. **(B)** Boxplots showing increased expression of ADH5 in the FA-predicted AML cells and in the FA non AML cells in comparison to healthy cells.
(JPEG)

S1 Table Gene list “Negative regulation of cell death”. (XLSX)

S2 Table Gene list “Regulation of the inflammatory response”. (XLSX)
