# Supplementary figures and images for: Prediction of myeloid malignant cells in Fanconi anemia using machine learning
Source: PLoS One. 2026 Jan 20;21(1):e0340578. doi: 10.1371/journal.pone.0340578 (PMC12818649; doi:10.1371/journal.pone.0340578)

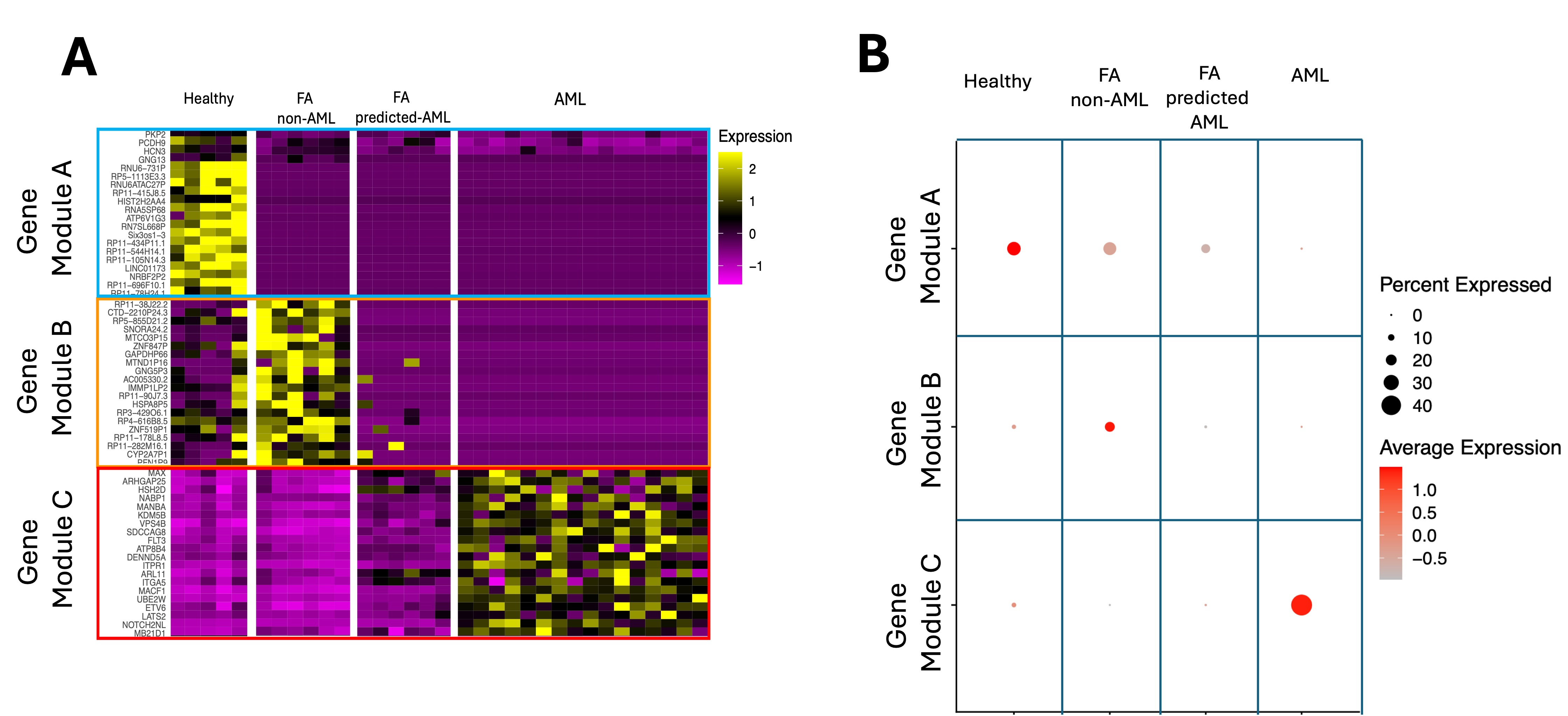

Supplement: S1 Fig — (A) Heatmap of differentially expressed genes, identified through pseudo-bulk analysis of the scRNAseq datasets, among healthy cells, FA non-AML cells, FA predicted-AML cells and AML cells. Genes that allow identification of cell types are classified in modules. (B) Module score analysis using scRNAseq data showing average expression of gene modules per cell type. (TIFF) [file pone.0340578.s001.tiff]

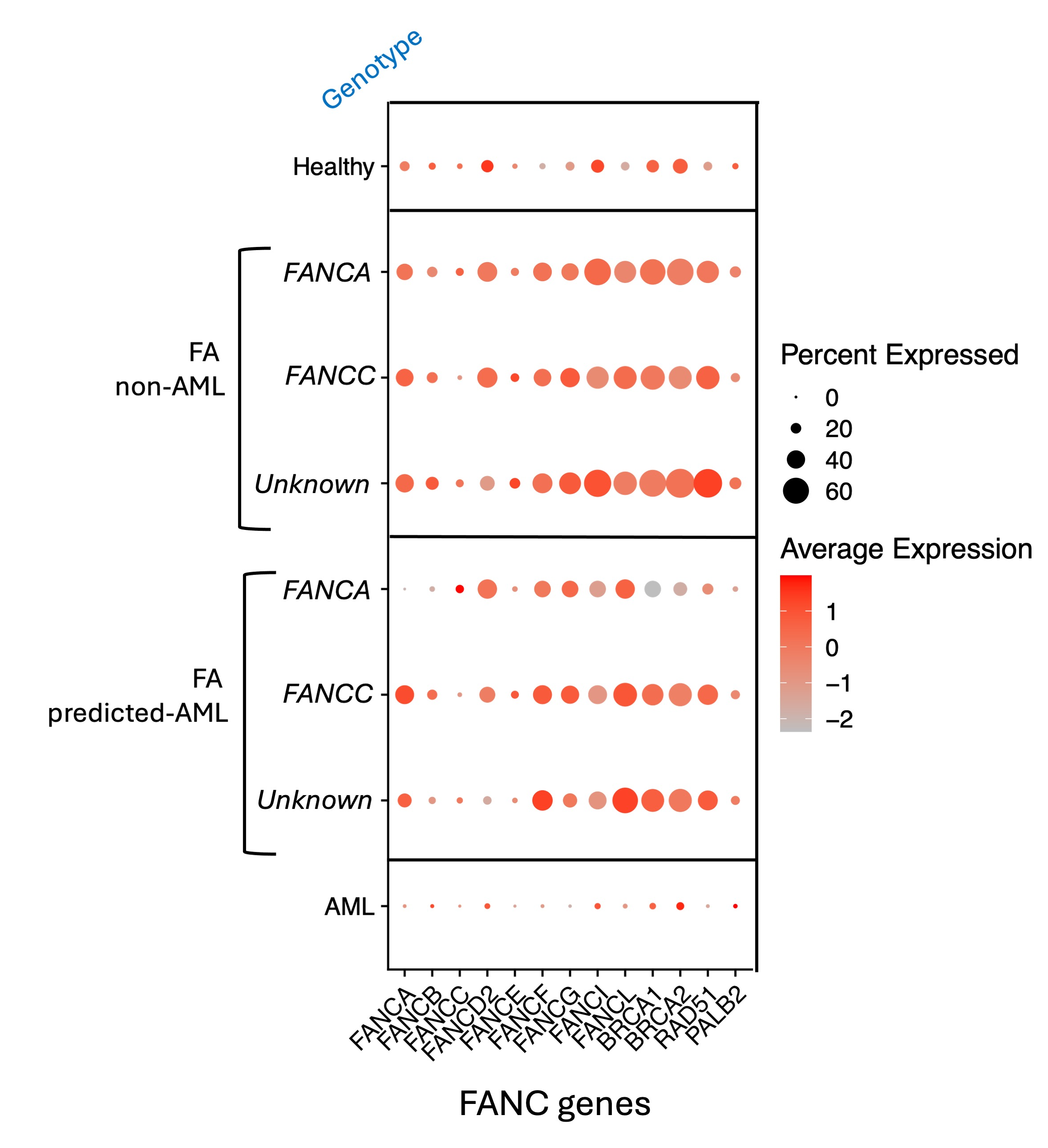

Supplement: S2 Fig — (A) Bubble plot showing the average expression of the FA pathway genes per cell type, dividing FA patients according to their germinal inactive gene. (TIFF) [file pone.0340578.s002.tiff]

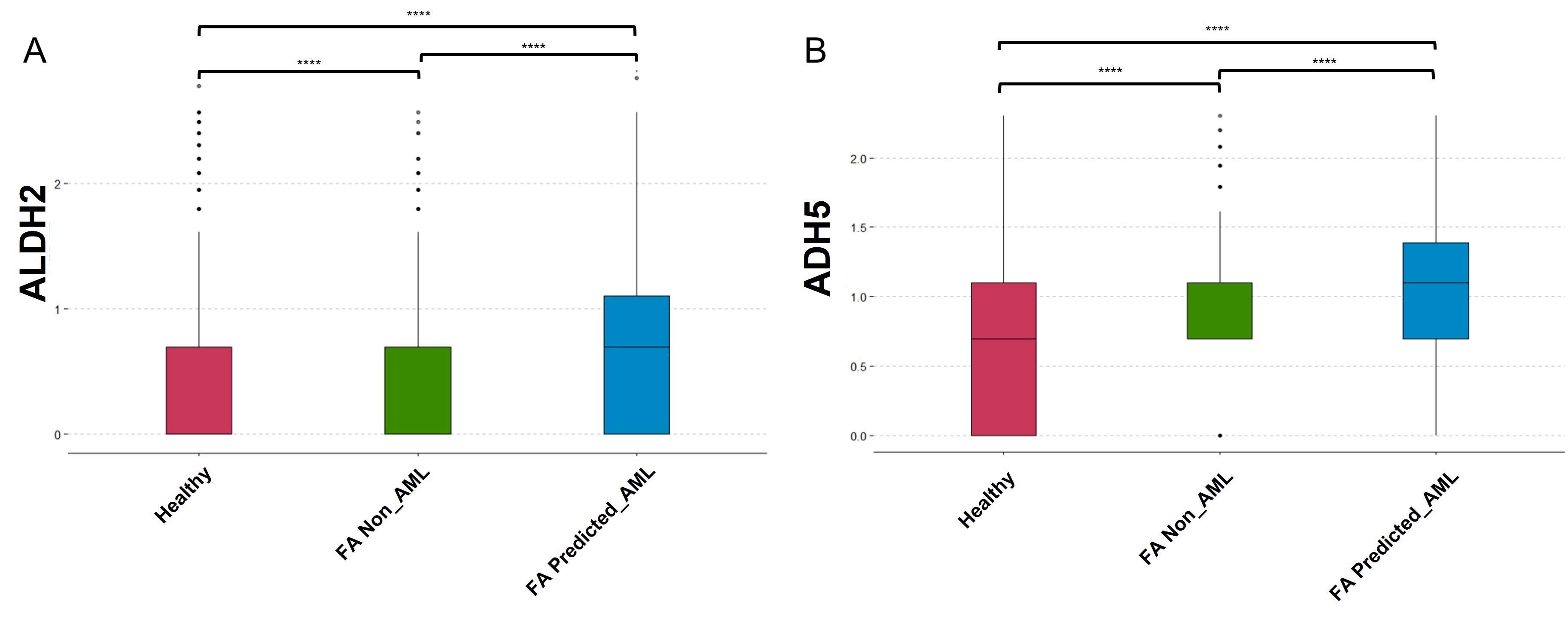

Supplement: S3 Fig — (A) Boxplots showing increased expression of ALDH1 in the FA-predicted AML cells in comparison to healthy and FA non AML cells. (B) Boxplots showing increased expression of ADH5 in the FA-predicted AML cells and in the FA non AML cells in comparison to healthy cells. (JPEG) [file pone.0340578.s003.jpeg]
